# The impact of head orientation with respect to B_0_ on diffusion tensor MRI measures

**DOI:** 10.1162/imag_a_00012

**Published:** 2023-09-25

**Authors:** Elena Kleban, Derek K. Jones, Chantal M.W. Tax

**Affiliations:** CUBRIC, School of Psychology, Cardiff University, Cardiff, United Kingdom; Inselspital, University of Bern, Bern, Switzerland; MMIHR, Faculty of Health Sciences, Australian Catholic University, Melbourne, Australia; CUBRIC, School of Physics and Astronomy, Cardiff University, Cardiff, United Kingdom; UMC Utrecht, Utrecht University, Utrecht, The Netherlands

**Keywords:** diffusion tensor imaging, magnetic resonance imaging, transverse relaxation, orientation anisotropy, fibre direction

## Abstract

Diffusion tensor MRI (DT-MRI) remains the most commonly used approach to characterise white matter (WM) anisotropy. However, DT estimates may be affected by tissue orientation w.r.t. ${\vec{B}}_{0}$ due to local gradients and intrinsic $T_{2}$ orientation dependence induced by the microstructure. This work aimed to investigate whether and how diffusion tensor MRI-derived measures depend on the orientation of the head with respect to the static magnetic field, ${\vec{B}}_{0}$. By simulating WM as two compartments, we demonstrated that compartmental $T_{2}$ anisotropy can induce the dependence of diffusion tensor measures on the angle between WM fibres and the magnetic field. In *in vivo* experiments, reduced radial diffusivity and increased axial diffusivity were observed in white matter fibres perpendicular to ${\vec{B}}_{0}$ compared to those parallel to ${\vec{B}}_{0}$. Fractional anisotropy varied by up to 20% as a function of the angle between WM fibres and the orientation of the main magnetic field. To conclude, fibre orientation w.r.t. ${\vec{B}}_{0}$ is responsible for up to 7% variance in diffusion tensor measures across the whole brain white matter from all subjects and head orientations. Fibre orientation w.r.t. ${\vec{B}}_{0}$ may introduce additional variance in clinical research studies using diffusion tensor imaging, particularly when it is difficult to control for (e.g., fetal or neonatal imaging, or when the trajectories of fibres change due to, e.g., space occupying lesions).

## 1 Introduction

MRI can provide invaluable information on tissue composition and structure *in vivo* through the manipulation of spins with magnetic fields. Several MRI contrasts have shown a dependence on tissue orientation w.r.t. the main magnetic field direction (${\vec{B}}_{0}$), including $T_{2}^{\left(*\right)}$, $T_{1}$, and magnetisation transfer (Bender & Klose, 2010; Cherubini et al., 2009; Denk et al., 2011; Gil et al., 2016; Knight et al., 2015, 2017, 2018; Lee et al., 2010, 2011; Oh et al., 2013; Pampel et al., 2015; Rudko et al., 2014; Sati et al., 2012, 2013; Schyboll et al., 2018, 2019; Wharton & Bowtell, 2012, 2013; Wiggins et al., 2008). Orientation dependence of the apparent $T_{2}^{\left(*\right)}$ in adult white matter (WM) has primarily been attributed to local magnetic susceptibility-induced gradients from the myelin sheath, and as such can provide valuable information on its condition in health and disease (Knight et al., 2018; Wharton & Bowtell, 2013). In addition, recent work (Bartels, Doucette, Birkl, Zhang, et al., 2022) found that orientational anisotropy of transverse relaxation rates in newborn WM, with a much lower degree of myelination, followed the pattern of residual dipolar coupling. Recent works have demonstrated different orientational behaviours of $T_{2}$-estimates in intra- and extra-axonal microstructural WM compartments (McKinnon & Jensen, 2019; Tax et al., 2021), see also Appendix A.

In diffusion MRI (dMRI), typically only the orientation dependence on externally applied spatial gradients is considered: It sensitises the signal to the diffusion of water molecules in one or multiple directions by deliberately applying magnetic field gradients, and as such can infer information on the directional organisation of tissue. At low to moderate diffusion weightings, the diffusion tensor MRI (DT-MRI) representation (Basser et al., 1994) remains the most commonly used approach to characterise the diffusion process, and DT-MRI-derived measures such as mean diffusivity (MD) and fractional anisotropy (FA) reflect both intra- and extra-axonal signal contributions.

Theoretically, dMRI signals and derived measures can also exhibit ${\vec{B}}_{0}$-orientation dependence when magnetic susceptibility variation is combined with anisotropic geometry at a subvoxel level. Several mechanisms may contribute to dMRI-signal anisotropy in this case. Firstly, several works have considered the interaction (or cross-term) of susceptibility-induced gradients with the externally applied diffusion encoding gradients, and their effect on estimates of the apparent diffusion coefficient (ADC) (Beaulieu & Allen, 1996; Clark et al., 1999; Does et al., 1999; Knight et al., 2017; Novikov, Reisert & Kiselev, 2018; Zhong & Gore, 1991; Zhong et al., 1991). Specifically, local gradients in the direction of the diffusion encoding gradient can lead to an under- or overestimation of ADC from individual isochromats, leading to a reduction of the overall ADC because isochromats with reduced ADC contribute a higher weighting (Zhong et al., 1991). By employing sequences sensitive and insensitive to local susceptibility-induced gradients, early *ex vivo* experiments in WM (Beaulieu & Allen, 1996; Trudeau et al., 1995) concluded that the effects from local gradients on diffusivity values did not have a measurable role in nerve samples at 4.7 T and 2.35 T, respectively, which was later corroborated *in vivo* at 1.5 T (Clark et al., 1999). Interestingly, Beaulieu and Allen (1996) did observe that diffusivity values along the axon varied by about 15% due to reorientation w.r.t. ${\vec{B}}_{0}$. *In silico* works provided theoretical background on the effect of mesoscopic susceptibility on ADC and DT-derived measures under variable diffusion times (Novikov, Reisert & Kiselev, 2018) and sample orientation (Knight et al., 2017), respectively. Furthermore, the recent observation of differences in compartmental $T_{2}$-anisotropy suggests another mechanism of ${\vec{B}}_{0}$-orientation dependence in DT measures. The intrinsic $T_{2}$-weighting of the diffusion-weighted spin-echo sequence affects the $T_{2}$-weighting of intra- and extra-axonal signal fractions. As a result, differences in compartmental $T_{2}$-orientation dependence w.r.t. ${\vec{B}}_{0}$ can lead to orientation-dependent variation in compartmental signal fractions and, consequently, affect DT measures.

This motivates further investigation of the potential orientational dependence of DT measure w.r.t. ${\vec{B}}_{0}$. The additional dMRI dependence on tissue orientation w.r.t. ${\vec{B}}_{0}$ may introduce variability in the results when not taken into account, potentially reducing statistical power to detect true effects, and could even provide important additional information on tissue microstructure (e.g., myelin). The aim of this work is to determine the variation of DT-MRI-derived measures as a function of fibre orientation w.r.t. ${\vec{B}}_{0}$. To this end, we investigate the effect of head-orientation dependence of compartmental $T_{2}$ (Tax et al., 2021) and the consequent variation of compartmental signal fractions on DT-MRI measures *in silico*, and characterise the ${\vec{B}}_{0}$-orientation dependence in *in vivo* human brain data at 3 T using a tiltable RF coil.

## 2 Methods

### 2.1 Simulations

The following simple simulations investigate the effect of ${\vec{B}}_{0}$-orientation dependence of compartmental $T_{2}$ (Tax et al., 2021) on estimated DT-MRI measures, thereby not considering cross-terms between the diffusion and background gradient. The simulations are based on a “standard model” of diffusion for white matter in the long-time limit, which models the intra-axonal space as a “stick” with zero perpendicular apparent diffusivity and the extra-axonal space as axially symmetric tensor (Assaf & Basser, 2005; Jespersen et al., 2007; Kroenke et al., 2004; Novikov et al., 2019). Different levels of complexity are investigated: First, in the case of no fibre dispersion and no noise, one can derive analytical equations for the ADC as a function of compartmental diffusivities, signal fractions, and compartmental $T_{2}$ (which can be ${\vec{B}}_{0}$-orientation dependent). Second, still in the case of no dispersion, the signal can be generated from analytical equations, noise added, and the DT fitted. Finally, this can be repeated for signals generated in the case of fibre dispersion.

For all simulations, scenarios for a range of θ (i.e., orientation w.r.t. ${\vec{B}}_{0}$) were generated corresponding to the distribution of θ observed in the *in vivo* data of all subjects and head orientations (see Section 2.2).

#### 2.1.1 Analytical case: no dispersion and no noise

Consider a simplified two-compartment model of the diffusion- and $T_{2}$ relaxation-weighted signal in WM (no fibre dispersion) as a function of the echo time, TE, and b-value:

$$S\left(b,g,\text{TE}\right)=f\cdot e^{-R_{2,\text{ia}}\text{TE}}\;\cdot e^{-bg^{T}D_{\text{ia}}g}\;+\left(1-f\right)\cdot e^{-R_{2,\text{ea}}\text{TE}}\;\cdot e^{-b\;g^{T}D_{\text{ea}}g},$$ (1)

where subscripts i/e denote intra-/extra-axonal compartments, respectively, $R_{2}=1/T_{2}$ are the relaxation rates, D are positive semi-definite diffusion tensors, and f is the intra-axonal signal fraction. Suppose $D_{\text{ia}}$ and $D_{\text{ea}}$ have equal principal eigenvectors (denoted by n) and parallel and perpendicular eigenvalues $D_{\text{ia},∥},D_{\text{ia},⊥}$ (where $D_{\text{ia},⊥}=0$) and $D_{\text{ea},∥},D_{\text{ea},⊥}$ respectively, then the signal can be simplified as

$$S\left(b,g,\text{TE}\right)\;˜\;f\cdot e^{-R_{2,\text{ia}}\text{TE}}\cdot e^{-bD_{\text{ia}}}+\left(1-f\right)\cdot e^{-R_{2,\text{ea}}\text{TE}}\cdot e^{-bD_{\text{ea}}},$$ (2)

where $D=D_{⊥}+{\left(g\cdot n\right)}^{2}(D_{∥}-D_{⊥})$.

Considering DTI as a signal representation at sufficiently low b-values, i.e., capturing the first order b-term in the Cumulant expansion (Jensen et al., 2005), one can derive expressions for the ADC, e.g., by expanding in powers of b the analytic expression for lnS(b) (Eq. 2). For non-interacting compartments, the diffusion coefficient is a weighted sum of the diffusivities in the individual compartments where the signal fractions are $T_{2}$-weighted. Specifically, the ADC is the first order term of the Maclaurin series expansion of ln(S) in b:

$$\text{ADC}\left(\text{TE}\right)=\frac{f\cdot D_{\text{ia}}\cdot e^{-R_{2,\text{ia}}\text{TE}}+\left(1-f\right)\cdot D_{\text{ea}}\cdot e^{-R_{2,\text{ea}}\text{TE}}}{f\cdot e^{-R_{2,\text{ia}}\text{TE}}+\left(1-f\right)\cdot e^{-R_{2,\text{ea}}\text{TE}}}.$$ (3)

Equation 3 was used to compute apparent axial diffusivity (AD, g∥n), radial diffusivity (RD, g⊥n), MD, and FA. Recent work suggests that the effect of WM fibre orientation θ on the magnetic field can most prominently be observed in the extra-axonal apparent transversal relaxation rate $R_{2,\text{ea}}=1/T_{2\text{ea}}$ (Tax et al., 2021). The dependence could be described as

$$R_{2,\text{ea}}\left(\theta \right)=R_{2,\text{iso}}+R_{2,\text{aniso}}{\text{sin}}^{4}\theta .$$ (4)

This orientational dependence of $R_{2,\text{ea}}\left(\theta \right)$ will result in orientational dependence of the ADC in addition to a straightforward TE dependence.

Analytical noiseless scenarios were simulated using Equations 3 and 4. TEs were selected to match the *in vivo* acquisition (cf., Section 2.2.1). The axonal fraction was varied f=[0.1, 0.3, 0.5, 0.7, 0.9], and diffusivities D and relaxation rates $R_{2}$ were set to the following values: $[D_{\text{i},∥},D_{\text{e},∥},D_{\text{e},⊥}]=[2.6,\;2,\;0.4]\;\;\mu {\text{m}}^{2}/\text{ms}$, and $R_{2,\text{i}}=12\;[s^{-1}]$, $R_{2,\text{e}}=17.4+2.4\cdot {\text{sin}}^{4}\theta \text{ [}s^{-1}\text{]}$, respectively.

#### 2.1.2 Noise simulations without dispersion

Equation 2 was used to simulate signals with TE, b and g matching the *in vivo* data Section 2.2. Signals were simulated using the same fractions, intra- and extra-axonal diffusivities, and relaxation rates as for the analytical simulations. Rician noise was added to the signal with an SNR of 100 on the b=0, TE=0 signal, similar to the *in vivo* acquisitions (Tax et al., 2021). DT were estimated for each TE on $b\leq 1500\;\text{s}/{\text{mm}}^{2}$ data using iterative weighted linear least squares, and AD, RD, MD, and FA were computed.

#### 2.1.3 Noise simulations with dispersion

Finally, the effect of fibre orientation dispersion was studied by forward simulating a distribution of orientation-dispersed compartments according to a Watson distribution, where each sub-compartment (i.e., each distinctly oriented extra-axonal compartment) can separately exhibit $R_{2}$-orientation dependence (Tax et al., 2021; Appendix A). Tissue properties, noise, and estimation were as described in the simulations without dispersion.

#### 2.1.4 Data analysis

To quantify the magnitude of orientation dependence, B, the simulated values of each DTI-derived measure at each TE were directly represented by a function of θ:

$$F\left(\theta \right)=A+B\cdot {\text{sin}}^{4}\theta .$$ (5)

We note that this representation does not exactly describe the orientation dependence even in the simplest analytical case (Eq. 3), but nevertheless provides a close approximation (see an example of a ${\text{sin}}^{4}\theta$-fitting in Figure S1) and allows for the quantification of anisotropy through the estimation of B. The performance of the anisotropic representation relative to the isotropic case, F(θ)=A, was estimated using the rescaled Akaike's Information Criterion (AIC) (Akaike, 1974; Burnham & Anderson, 2004): $\Delta_{\text{AIC}}=\text{AIC}-{\text{AIC}}_{\text{min}}$. Here, ${\text{AIC}}_{\text{min}}$ is the minimal AIC value in the set. Per (Burnham & Anderson, 2004), $\Delta_{\text{AIC}}$ values allow a comparison of the relative merits of representations in the set as follows: Representations having $\Delta_{\text{AIC}}\leq 2$ are considered to have similar substantial support as the representation with ${\text{AIC}}_{\text{min}}$, those with $4\leq \Delta_{\text{AIC}}\leq 7$ have considerably less evidence, and those with $\Delta_{\text{AIC}}\geq 10$ have no support. Additionally, the isotropic model is selected over the anisotropic, if the 85% confidence interval of the magnitude of anisotropy included zero (Arnold, 2010).

### 2.2 In vivo data

In this work, we used a subset of the multi-dimensional diffusion-$R_{2}$ data presented in previous work (Tax et al., 2021), and relevant data acquisition and pre-processing steps are re-iterated below. The study was approved by the Cardiff University School of Psychology Ethics Committee, and written informed consent was obtained from all participants in the study.

#### 2.2.1 Data acquisition

**Fig. 1.**
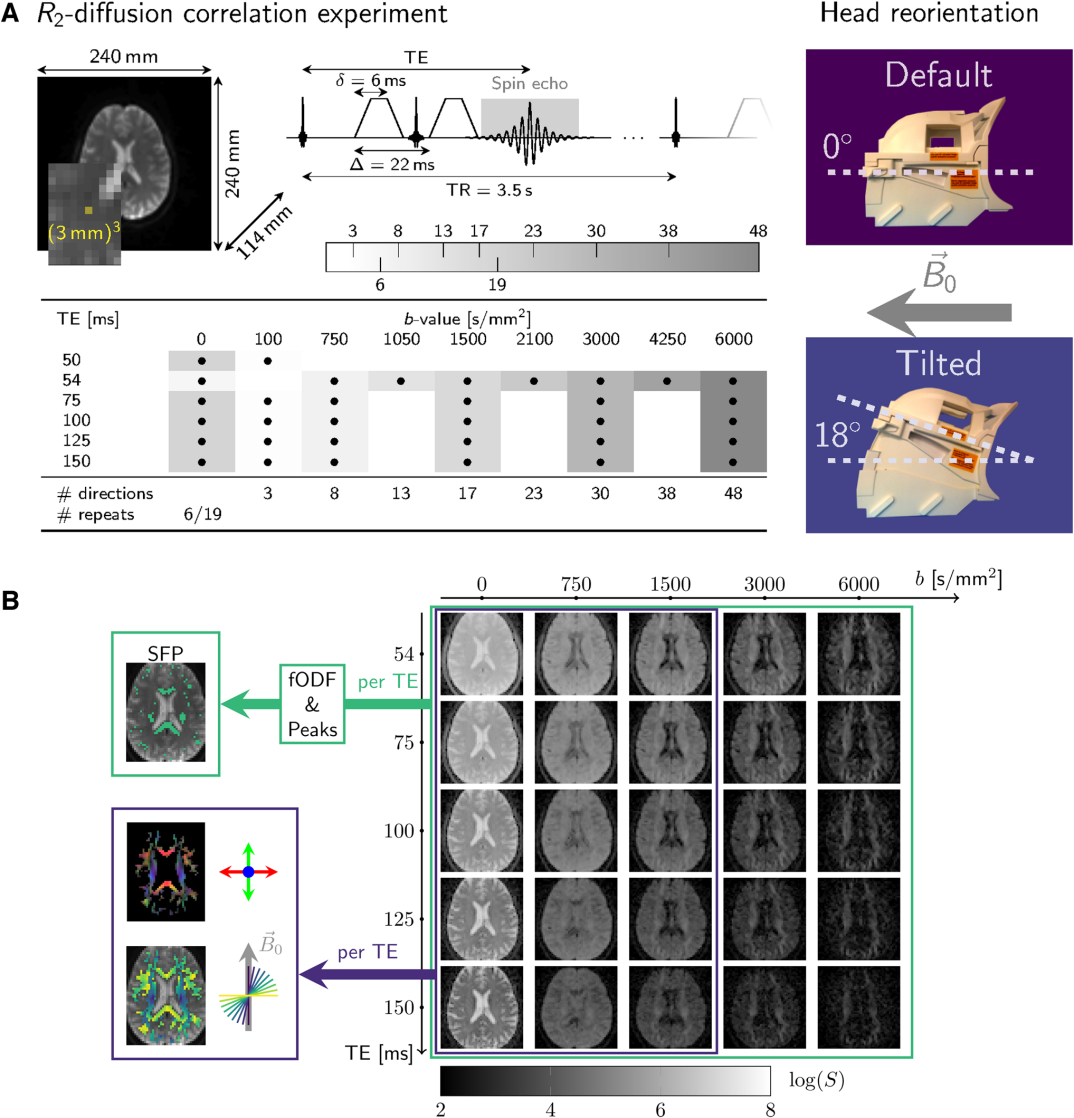
Methods. (A) Multi-dimensional $R_{2}$-diffusion data were acquired under simultaneous modulation of echo times and diffusion-gradient amplitudes in a pulsed-gradient spin-echo sequence with EPI readout. Time between diffusion gradients, Δ=22 ms, and diffusion gradient duration, δ=8 ms, were kept fixed for all echo times. The gradient orientations were defined in scanner coordinates and thus were not rotated with the head re-orientation. Additional modulation of fibre orientation was achieved by head re-orientations relative to ${\vec{B}}_{0}$. (B) A subset of the pre-processed multi-dimensional diffusion-$R_{2}$-weighted dataset from previous work (Tax et al., 2021) was used to calculate echo-time-dependent diffusion tensors and fibre orientation to $B_{0}$ (denoted by θ) (blue, bottom left), and single-fibre-population (SFP) voxels (green, top left).

Multi-dimensional diffusion-$R_{2}$-weighted data were acquired from five healthy participants (3 female, 25-31 y.o.) on a 3 T MRI scanner equipped with a 300 mT/m gradient system and a 20ch head/neck receive coil that can tilt about the L-R axis (Siemens Healthineers, Erlangen, Germany). The acquisition was repeated in default ($0^{\circ }$) and tilted ($18^{\circ }$) coil-orientation to introduce variable anatomical orientation w.r.t. ${\vec{B}}_{0}$. Acquisition parameters are summarised in Figure 1A.

#### 2.2.2 Data processing

The data were checked for slice-wise outliers (Sairanen et al., 2018) and signal drift, corrected for Gibbs ringing (Kellner et al., 2016), subject motion, geometrical distortions (Andersson & Sotiropoulos, 2016; Andersson et al., 2003; Glasser et al., 2013), and noise bias (Koay, Ozarslan, & Basser, 2009; Koay, Özarslan, & Pierpaoli, 2009; St-Jean et al., 2016, 2020).

From the pre-processed data, a subset with diffusion weightings matching across echo times was selected (Fig. 1B), and for each echo time diffusion tensors, fibre orientation w.r.t. ${\vec{B}}_{0}$ and single fibre population masks were obtained as described below. DT were estimated for each TE on the nominal $b=\left[0,\;\;750,\;\;1500\right]\text{s}/{\text{mm}}^{2}$ data, using iterative weighted linear least squares. Gradient non-linearities were considered and b-values/-vectors were corrected correspondingly prior to fitting (Rudrapatna et al., 2021). Fibre orientations θ w.r.t. ${\vec{B}}_{0}$ were computed from the first eigenvector of the estimated DT. Note that ${\vec{B}}_{0}$ has to be in image coordinates of each subject/head orientation.

Fibre orientation distribution functions (fODF) (Descoteaux et al., 2008; Tournier et al., 2007) were estimated per TE using multi-shell multi-tissue constrained spherical deconvolution (Jeurissen et al., 2014) from the data acquired at TE=54 ms. From the fODFs, single-fibre population (SFP) voxels with low dispersion ($p_{2}>0.5$) were identified (Tax et al., 2014). Dispersion was quantified by $p_{2}=\sqrt{4\pi /5}\sqrt{\sum_{m}\;\;|p_{2m}{|}^{2}}$, where p are spherical harmonics coefficients (Novikov, Veraart, et al., 2018; Reisert et al., 2017; Tax et al., 2021).

We used WM tract segments extracted in previous work (Tax et al., 2021). Briefly, 18 major WM tracts and, where applicable, their bilateral counterparts were extracted and segmented using TractSeg (Wasserthal et al., 2018).

#### 2.2.3 Data analysis

##### θ -dependence of DT measures: pooling all SFP voxels

General trends in orientational anisotropy of DTI measures were investigated by subdividing the range of angles into bins, averaging the estimates within each bin, and smoothing. Specifically, the data were binned in $1^{\circ }$-subsets and the corresponding DT-measure estimates and θ-values were averaged across each bin, denoted as ${\langle \text{measure}\rangle }_{1^{\circ }}$ and ${\langle \theta \rangle }_{1^{\circ }}$. Then, a smoothing spline as a function of ${\langle \theta \rangle }_{1^{\circ }}$ and weighted by the number of data points in each bin was fitted to ${\langle \text{measure}\rangle }_{1^{\circ }}({\langle \theta \rangle }_{1^{\circ }})$. An example of this procedure is shown in Figure S2 for the lowest TE.

The magnitude of anisotropy was defined as the difference between the minimal and the maximal values of the fitted curves. Their signs were set negative if the minimal values were below those at θ=0. The contribution of orientational anisotropy to overall variance was calculated as: $({\text{std}}_{\text{iso}}-{\text{std}}_{\text{aniso}})/{\text{std}}_{\text{iso}}$. Here, ${\text{std}}_{\text{aniso}}$ and ${\text{std}}_{\text{iso}}$ are the standard deviations across all SFP voxels with and without orientational anisotropy being considered, respectively. Additionally, mean values A across all SFP voxels were obtained for each measure and TE.

In addition to the spline analysis, to assess whether DT measures as a function of θ showed significant orientation dependence, we assessed whether an anisotropic representation described the data better than isotropic (cf., Supporting Information), using the approach similar to the *in silico* analysis described in Section 2.1.

##### θ -dependence of DT measures: tractometry analysis to achieve spatial correspondence

By comparing the measures estimated within the same anatomical region at default or tilted coil orientation, we aimed to reduce the effects of the potential microstructural variability across the WM in the approach described above. The anatomical correspondence between the coil-orientations was established using the segments derived from the tractometry approach. The outer-most 20% of tract segments and the segments with 3 or fewer voxels were excluded to minimise the effects of fanning and noise, respectively. To obtain the effect of the re-orientation, we evaluated ${\langle {\text{measure}}_{0^{\circ }}\rangle }^{\text{s}}-{\langle {\text{measure}}_{18^{\circ }}\rangle }^{\text{s}}$ as a function of ${\langle {\text{sin}}^{4}\theta_{0^{\circ }}\rangle }^{\text{s}}-{\langle {\text{sin}}^{4}\theta_{18^{\circ }}\rangle }^{\text{s}}$. Here, ${\langle \rangle }^{\text{s}}$ denotes the average of corresponding values from SFP voxels over each segment, and the subscripts $0^{\circ }$ and $18^{\circ }$ correspond to default and tilted head orientations, respectively.

## 3 Results

### 3.1 Simulations

**Fig. 2.**
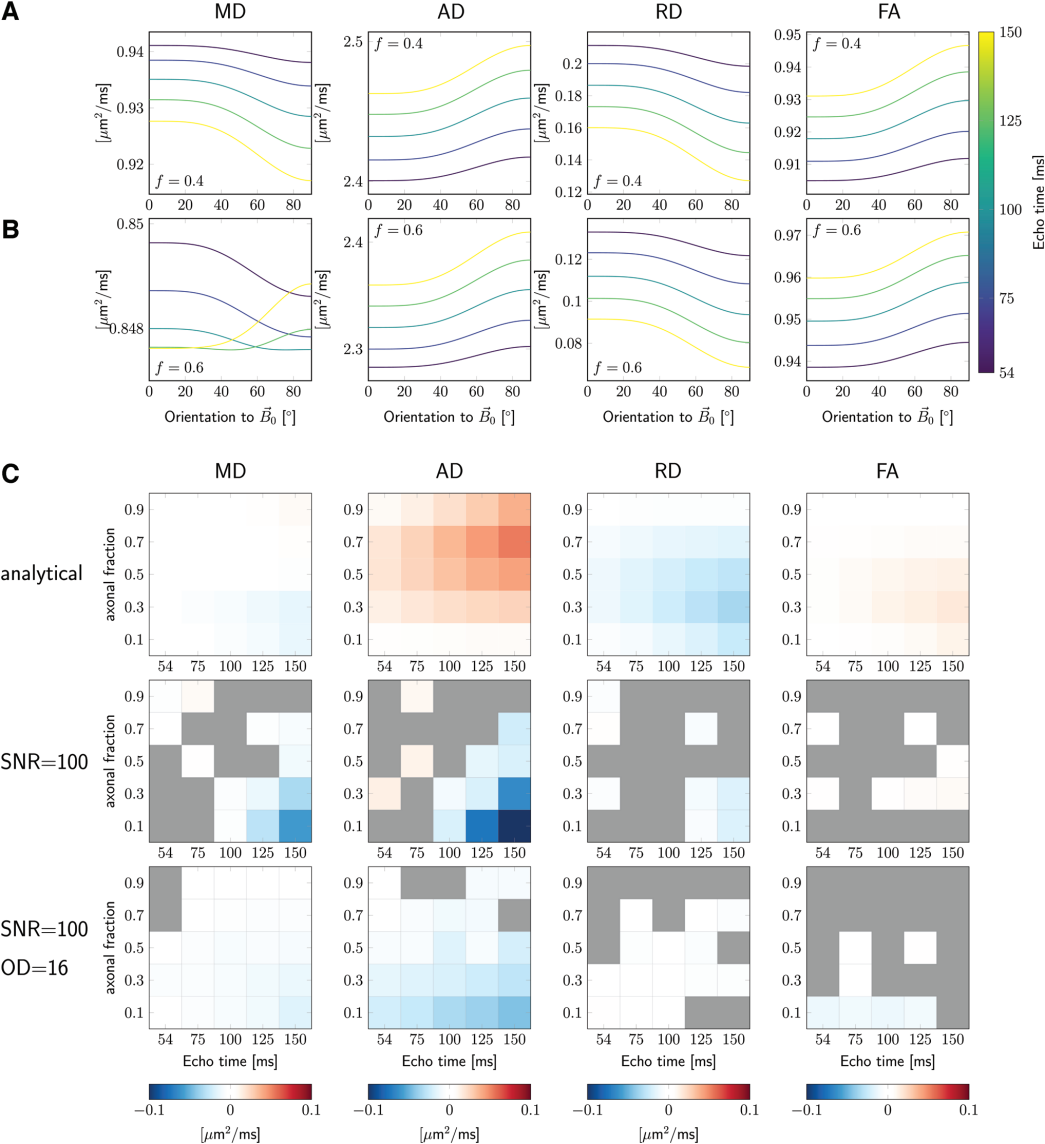
Simulation results. The signals were estimated for variable echo times and axonal fractions, f, and fixed diffusivities $[D_{\text{i},∥},D_{\text{e},∥},D_{\text{e},⊥}]=[2.6,\;2,\;0.4]\;\;\mu {\text{m}}^{2}/\text{ms}$, and relaxation rates $R_{2,\text{i}}=12$, $R_{2,\text{e}}=17.4+2.4\cdot {\text{sin}}^{4}\theta$. Figures in (A) and (B) were estimated analytically (Eqs. 3 and 4) and show MD, AD, RD, and FA as functions of fibre orientation w.r.t. ${\vec{B}}_{0}$ for f=0.4, and f=0.6, respectively. In (C) the magnitude of anisotropy, B, (colors) is shown as a bi-modal function of the echo time (horizontal axis) and the axonal fraction (vertical axis, f=[0.1,  0.3,  0.5, 0.7, 0.9]). Columns left-to-right are different DTI measures: MD, AD, RD, and FA; rows top-to-bottom are different simulation conditions: using the analytical expression, assuming noisy signal with SNR=100, and adding fibre dispersion (OD=16) in addition to noise, respectively.

Figure 2A and B shows examples of MD, AD, RD, and FA as a function of fibre orientation θ to ${\vec{B}}_{0}$ for the noiseless analytical simulations without fibre dispersion. For the parameter settings investigated, AD and FA increase with θ (the magnitude of anisotropy, $\hat{B}>0$), while RD decreases ($\hat{B}<0$). The absolute value of the magnitude of anisotropy, $\left|\hat{B}\right|$, generally increases with TE. The resulting behaviour of MD is non-trivial and sensitive to simulation parameters (e.g., axonal signal fraction f), with possible sign flips of $\hat{B}$ for increasing TE.

Figure 2C shows results for the analytical simulations following Equations 3 and 4 (first row), and the noisy simulations without (middle row) and with (third row) fibre dispersion. The plots show the estimated anisotropy $\hat{B}$ (colormap) for the scenario $D_{∥,\text{i}}=2.6\mu {\text{m}}^{2}/\text{m}s$, $D_{∥,\text{e}}=2\mu {\text{m}}^{2}/\text{ms}$, and $D_{⊥,\text{e}}=0.4\mu {\text{m}}^{2}/\text{ms}$, echo times matching the acquisition parameters (horizontal axis) and a range of f (vertical axis). The columns show results for different DT measures. A grey colour indicates scenarios for which an isotropic representation was favoured (Section 2.2.3). It becomes immediately apparent that the effect on DT measures can be vastly different depending on the scenario: In the simple analytical simulations, B for MD can be either positive (high f) or negative (low f) depending on the intra-axonal signal fraction and its absolute value becomes larger for increasing TE. For the simulation with noise and no dispersion, B can be positive or negative, and in the case of dispersion B is lower and negative in the cases investigated. For AD, B is predominantly positive in the non-dispersion analytical scenario and has the largest value for high TE, but in the noisy simulations B could be negative. The behaviour of $B_{\text{RD}}$ is more consistent across simulation scenarios. Whereas $B_{\text{FA}}$ is mostly positive and largest for high TE and low f in the no-dispersion noiseless and noisy cases, B can be positive or negative in the noisy scenarios but is overall low or non-significant.

### 3.2 In vivo data

#### 3.2.1 Pooled data

**Fig. 3.**
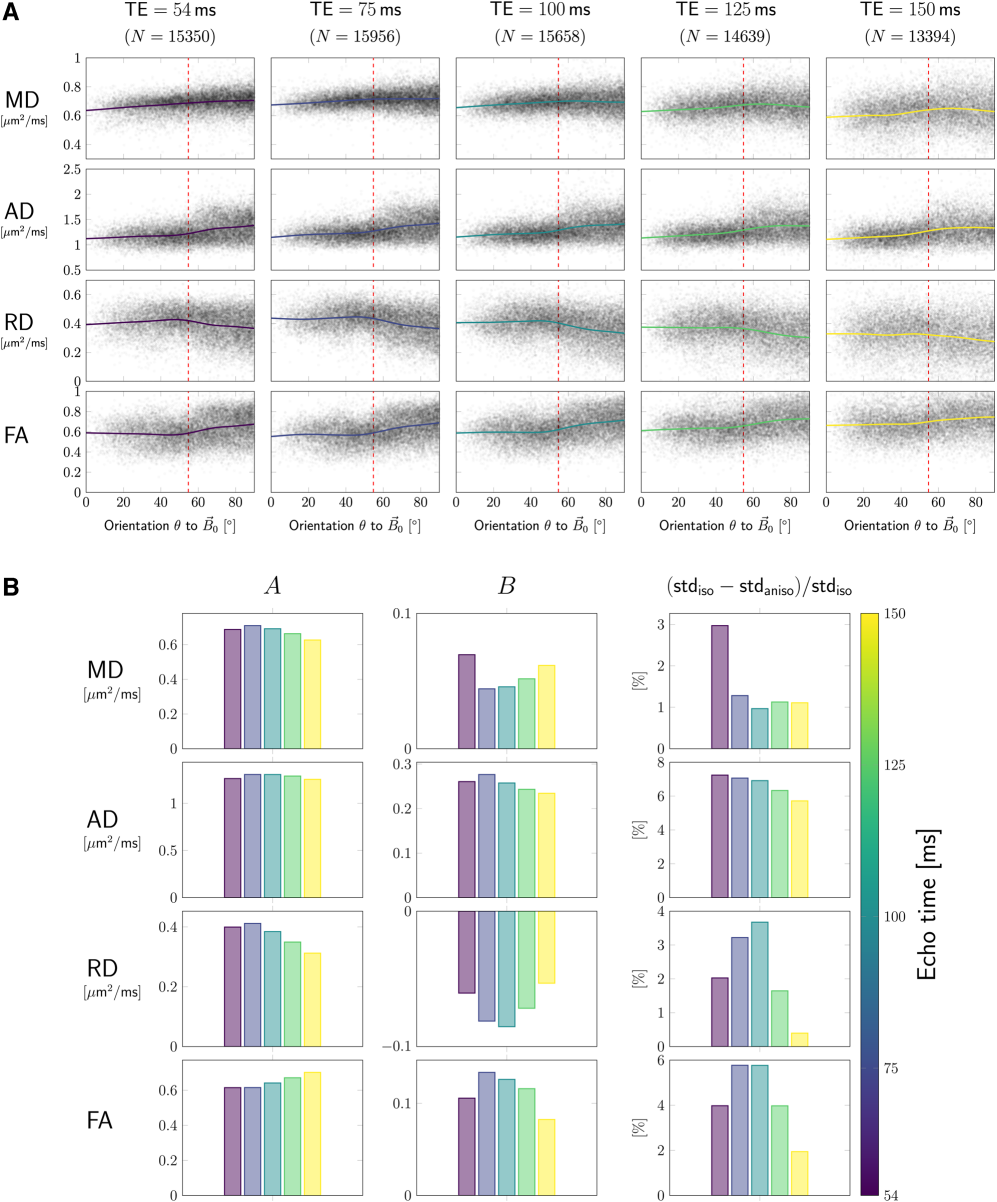
Pooled SFP data results. (A) Each DTI measure (rows) from SFP voxels was plotted against the fibre orientation, θ, to the magnetic field. Each column/colour corresponds to a different TE. Solid lines represent best fitting smoothing spline curves. Dashed red lines indicate the magic angle of $54.7^{\circ }$. (B) The estimated mean value, A, and the magnitude of anisotropy, B, over all SFP voxels are shown in the first and the second column, respectively. The third column shows the amount of decrease in variation of values when orientation w.r.t. ${\vec{B}}_{0}$ is taken into account. Colours represent the corresponding echo times, for which anisotropy of the measures was investigated.

In Figure 3A, DT measures are plotted as functions of fibre orientation θ w.r.t. ${\vec{B}}_{0}$ (horizontal axes), and echo time TE (columns), along with the corresponding smoothing spline curves highlighting anisotropic effects. The data were pooled from all subjects and both head orientations; each data point represents one SFP voxel. RD and FA show global maxima and minima, respectively, close to the magic angle (dashed red lines), most prominently for low TE.

The barplots in Figure 3B show the average value (A, first column) or the magnitude of anisotropy (B, second column) obtained from all SFP voxels for a given measure (rows). MD, AD, and FA increase as a function of θ (B>0), while RD decreases (B<0). The anisotropic component B is least dependent on the echo time for axial diffusivity. For other measures, B(TE) is non-monotonic (for evaluated TE-s) with its absolute value being minimal (for MD) or maximal (RD, FA) at around 75-100 ms. The fibre-orientation-independent component A (first column, Fig. 3B) evolves non-monotonically as a function of TE. The relative range of change of DT measures across angles (computed as B/A, results not shown) can reach values up to 20%. Finally, column three of Figure 3B shows the fraction by which anisotropy effects contribute to overall variance, showing the largest contribution for AD (around 7% at TE=54,ms). For MD, RD, and FA, the variance contribution was 3%, 2%, and 4%, respectively, at the same shortest echo time.

We also observed an overall similar behaviour in magnitude of anisotropy B when the pooled data were evaluated using ${\text{sin}}^{4}\theta$-representation instead of the spline fit (cf., Fig. S3).

#### 3.2.2 Segment-wise comparison

**Fig. 4.**
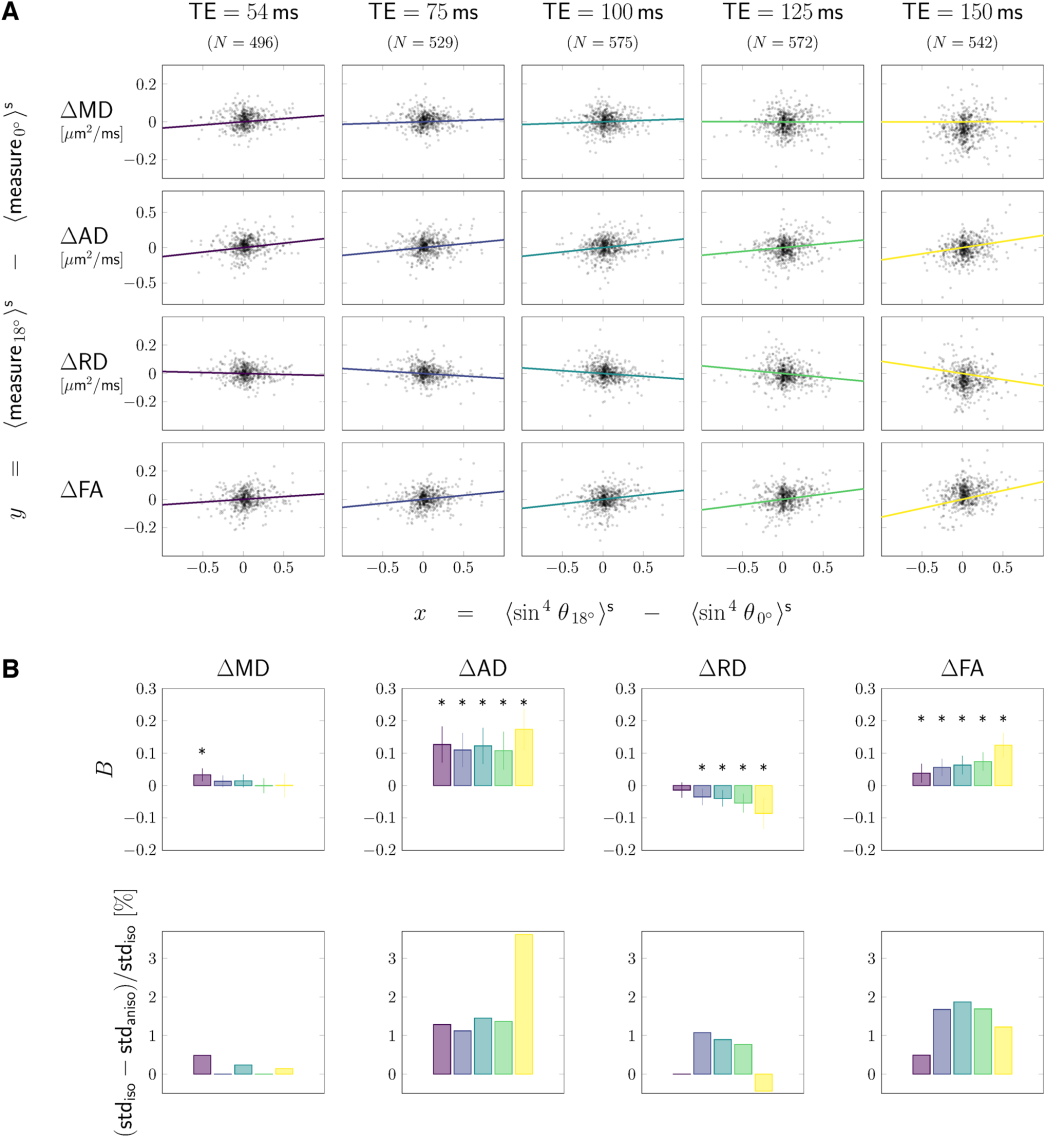
Tractometry results. Tractometry was used to achieve anatomical correspondence between tilted and default head orientation, by comparing values of DTI measures in default vs tilted head orientations tract- and segment-wise. (A) In scatterplots, changes in value of the respective DTI measure with re-orientation (rows) are plotted as a function of the corresponding change in ${\text{sin}}^{4}\theta$. (B) Barplots show: the magnitude of anisotropy estimated for each DTI measure and each echo time (top row); and the change in standard deviation (std) when fibre anisotropy is taken into account (bottom row). Data in which anisotropic representation (y=B⋅x) described the data better (${\text{AIC}}_{\text{iso}}-{\text{AIC}}_{\text{aniso}}>2$) than isotropic assumption (B=0) were indicated by a *-symbol.

The scatterplots in Figure 4A show segment-wise differences between the values in tilted and default head orientation of each measure (rows) against the ${\text{sin}}^{4}\theta$ of fibre orientations w.r.t. ${\vec{B}}_{0}$. Each column and the corresponding colour of the linear fit y=B⋅x represent different echo times. The fitting results are summarised in the top row of Figure 4B, and the fraction of variance contributed by the anisotropy effects is in the barplots of the bottom row.

Compared to the pooled analysis, the sign of anisotropy was the same (positive for MD, AD, and FA and negative for RD), but the trend as a function of TE was different for the segment-wise analysis (e.g., the magnitude of anisotropy |B| in RD increased with echo time whereas the pooled analysis showed a decrease for the largest echo times).

## 4 Discussion

We used diffusion-$T_{2}$-correlation data acquired in two head orientations using a tiltable coil (Tax et al., 2021) to achieve a larger range of orientations and investigate the effect of head orientation on diffusion tensor measures: mean, axial, and radial diffusivities, and fractional anisotropy. We observed that fibre orientation w.r.t. ${\vec{B}}_{0}$ may be responsible for up to three, seven, and two percent of variance in MD, AD, and RD, respectively, at TE=54
ms and about four percent of variance in FA at the same TE. We also utilised tractometry to achieve anatomical correspondence and used the ${\text{sin}}^{4}\theta$-representation to estimate the effect of head reorientation.

### 4.1 TE dependence of DTI measures

Echo-time dependence of diffusion coefficients and DT-derived measures has long been recognised. Does and Gore (2000) have reported an increase/decrease of ADC with longer TE when diffusion weighting was applied parallel/perpendicular to the rat's trigeminal nerve. These are in correspondence with analytical observations visualised in, e.g., Figure 2A: axial diffusivity, AD, increases, while radial diffusivity, RD, decreases with longer echo times. Assaf and Cohen (2000) performed diffusion experiments with variable echo time to demonstrate the presence of two distinct diffusing compartments; they also found that the signal of the slow diffusing component has a lower $R_{2}$ relaxation rate. This, again, would correspond to the decrease in radial diffusivity with longer TE. Finally, Qin et al. (2009) have explored DTI measures as functions of echo time in the rhesus monkey internal capsule. They similarly reported a decrease in the radial and an increase in axial diffusivities with longer TEs, but also an increase in fractional anisotropy and no significant changes to the mean diffusivity. Lin et al. (2018) made similar observations for the human corpus callosum and internal capsule; in addition, they observed no TE dependence of AD in the corpus callosum.

In our data, which were pooled from WM SFP voxels, we did not observe any linear trends (cf. isotropic representation, A, Fig. 3B); the non-monotonic variations of the DTI measures could be due to the variability of each measure as a function of TE between SFP voxels. Additionally, the much noisier data at longer TEs could also have contributed to these differences. Yet, for echo times ≥75
ms, we observed a decrease in RD and an increase in FA, which agree with observations made by Qin et al. (2009) and Lin et al. (2018), and similar to the latter we saw no significant changes in AD.

From the same data, compartmental transverse relaxation rates were previously estimated (Tax et al., 2021), and faster extra-axonal signal decay was observed, which is in correspondence with previous findings (Assaf & Cohen, 2000; Does & Gore, 2000; Peled et al., 1999; Veraart et al., 2018).

### 4.2 Head-orientation dependence of DT measures

#### 4.2.1 Orientational anisotropy of DT measures observed in vivo and in silico

We estimated non-zero magnitude of orientation anisotropy in all DTI measures with both methods: pooled SFP voxels, and tract-segment-wise comparison between default and tilted head orientations. Under the assumption of ${\text{sin}}^{4}\theta$-behaviour, the correspondence in estimated magnitude of anisotropy between the two methods was higher at shorter echo times of 54
ms and 75
ms, and the accuracy at longer echo times was potentially compromised by decreased SNR. Similarly, the contribution of anisotropy effects to the variance of DTI measures decreased with increasing echo time. Comparing the spline with the ${\text{sin}}^{4}\theta$-representation in the pooled results, the absolute values of B obtained using spline fitting were subtly higher than those estimated using the ${\text{sin}}^{4}\theta$-approximation, but overall followed the same trend as a function of TE.

The *in vivo* RD and MD estimates as a function of θ followed trends also seen in the analytical simulations, i.e., positive B for AD and FA and negative B for RD. However, also opposite signs for B were observed in the noisy simulations, e.g., in AD. This could not merely be caused by $D_{\text{e},∥}>D_{\text{i},∥}$ (the opposite was simulated), but it is hypothesised that this could be attributed to the complexity of tissue (e.g., dispersion, a distribution of diffusivities and $T_{2}$ within and across voxels in the *in vivo* results, and other origins of orientation dependence) and different levels of noise, amongst others. One can also observe that the estimated B of AD decreased as a function of TE*in vivo* in contrast to the increase in the toy-example.

#### 4.2.2 Origin of anisotropic effects of DTI in WM

The simulations considered the effect of $T_{2}$-weighting and different $T_{2}$-anisotropy behaviour in the intra- and extra-axonal space on DT measures, assuming a dominant role for myelin susceptibility effects in the extra-axonal space. However, the origin of the orientation dependence may be more complex. In *in vivo* data, both the ${\text{sin}}^{4}\theta$ behaviour and a more general spline representation were used to investigate the θ-dependence, indeed resulting in a similar estimated contribution of orientation dependence to the variance of DT measures in the pooled analysis and similar magnitudes of anisotropy. This similarity partially supported the assumption made in simulations, i.e., that the difference in $R_{2}(\theta )$-dependence between the intra- and extra-axonal signals (i.e. ${\text{sin}}^{4}\theta$-dependence in the extra-axonal space) is a major contributor to the orientational anisotropy. Yet, the behaviour of the spline curves deviates from the typical ${\text{sin}}^{4}\theta$-shape, which indeed suggests that the nature of anisotropy must be more complex.

The hypothesis that self-induced gradients arising from local variations in magnetic susceptibility could be an additional source of variation in apparent diffusion coefficients has been proposed by several works (Beaulieu & Allen, 1996; Trudeau et al., 1995). Trudeau et al. (1995) measured diffusivity values at 4.7 T in excised porcine spinal cords at room temperature, with diffusion gradients applied parallel and perpendicular to the primary fibre orientation. By reorienting the sample relative to the main magnetic field direction, they were able to manipulate the distribution of local magnetic susceptibility. Beaulieu and Allen (1996) performed similar experiments at 2.35 T on excised nerve fibres from garfish and frog. Both studies reported no detectable impact of local gradients on diffusivity values in these samples, and neither attributed the observed (Beaulieu & Allen, 1996) orientation dependence w.r.t. ${\vec{B}}_{0}$ to the effects of local gradients. Upon closer inspection of Trudeau et al. (1995; Fig. 3), a trend may be apparent with regards to fibre orientation w.r.t. ${\vec{B}}_{0}$. Although the distributions of $D_{∥}$- or $D_{⊥}$-values overlap when measured at either sample orientation, the average values for $D_{∥}$ seem lower and the average values for $D_{⊥}$ seem higher when the primary fibre orientation is along ${\vec{B}}_{0}$. Beaulieu and Allen (1996) solidified the apparent trend for the dependence of $D_{∥}$-values on primary fibre orientation w.r.t. ${\vec{B}}_{0}$, by reporting 15% lower values measured when fibres were along the magnetic field. Similarly, in our *in vivo* data, axial diffusivities were higher for fibres across ${\vec{B}}_{0}$ compared to fibres along ${\vec{B}}_{0}$, while radial diffusivities followed the opposite trend. Knight et al. (2017) have previously simulated the effects of mesoscopic magnetic field inhomogeneities near a hollow cylinder on $T_{2}$ and also reported head-orientation dependence of MD and FA values. They considered cross-terms between local gradients and encoding gradients to be negligible. Wang et al. (2022) have rotated an extracted mouse brain w.r.t. the main magnetic field and evaluated MD and FA for seven major brain regions, one of which was white matter. They did not observe any significant variations across orientations of MD/FA in WM; however, they did not break down WM into sub-ROIs of similar fibre orientation, potentially averaging away effects due to re-orientation. Bartels, Doucette, Birkl, Weber, et al. (2022) have recently studied MD, AD, and RD as a function of fibre orientation w.r.t. ${\vec{B}}_{0}$. They reported MD to behave in correspondence with simulations by Knight et al. (2017), but AD/RD obtained from their data are respectively minimal/maximal around the magic angle, suggesting a different origin of anisotropy. Interestingly, our data showed similar trends (cf., spline curves or piecewise average in SI). RD also showed a local maximum near the magic angle. The AD-curves appeared monotonous but still an increase in gradient around the same angle was evident. Additionally, a local minimum was apparent in the FA-curves. Pang (2022, 2023) also suggests an important role for magic angle effects.

Studies which investigate the ${\vec{B}}_{0}$-related anisotropic effects in DTI are limited in number. Yet, the anisotropic effects in DTI measures from WM observed here are coherent with those seen in previous works investigating $R_{2}^{\left(*\right)}$-anisotropy, though comparatively less pronounced. The majority of studies cover anisotropic effects of the WM signal evolution from the multi-echo gradient-recalled-echo (mGRE) sequence (Bender & Klose, 2010; Cherubini et al., 2009; Denk et al., 2011; Lee et al., 2010., 2011; Oh et al., 2013; Rudko et al., 2014; Sati et al., 2012, 2013; Wharton & Bowtell, 2012, 2013; Wiggins et al., 2008): Thanks to its sensitivity to $B_{0}$-inhomogeneities, it provides strong contrast in regions composed of tissues with different magnetic susceptibilities (myelinated WM fibres, in this particular case). Although most dMRI sequences are spin-echo-based, in which the $B_{0}$-effects are refocused, some magnetic susceptibility effects may shine through. On the one hand, incoherent molecular motion happening between the excitation pulse and the spin-echo combined with local $B_{0}$-inhomogeneities induced by the myelin sheath may lead to residual non-fully-refocused phases; on the other hand, echo-planar readout has some unavoidable $R_{2}^{\text{*}}$-weighting during the acquisition window. That said, the centre of the k-space is closer to the centre spin-echo, and is therefore less affected; additionally, lower-resolution data are expected to suffer less from this effect. Indeed, Gil et al. (2016) reported ${\text{sin}}^{4}\theta$-dependence of macroscopic $R_{2}$-values on fibre orientation θ to ${\vec{B}}_{0}$.

Another candidate for the orientational dependence of $R_{2}$ is the aforementioned magic angle effect (or dipole-dipole interactions) with the characteristic ${\left(3{\text{cos}}^{2}\theta -1\right)}^{2}$-behaviour. So far, those were not only considered the primary source of WM $R_{2}$-anisotropy in adults in the *in vivo* and postmortem brain, but also not excluded as a potential contributor (Birkl et al., 2020; Lee et al., 2011; Oh et al., 2013). Interestingly, Bartels, Doucette, Birkl, Zhang, et al. (2022) studied $R_{2}$ orientation dependence in the newborn brain having low myelination and observed very different behaviour from the adult brain, suggesting a primary role for residual dipolar coupling. In the absence of myelin, neurofilaments and microtubules of the axonal cytoskeleton are aligned with the axon and are hypothesised to contribute to orientation dependence. Similar observations were made on our data separating the intra- and extra-axonal relaxation rates (Tax et al., 2021): $R_{2}\left(\theta \right)˜{\left(3{\text{cos}}^{2}\theta -1\right)}^{2}$ fitted the intra-axonal data best.

Summarising, compartmental $R_{2}^{\left(*\right)}$-values have been reported to depend on orientation differentially (Kleban et al., 2020; Sati et al., 2013; Tax et al., 2021; Wharton & Bowtell, 2012), which could intrinsically lead to DT dependence on fibre orientation w.r.t. ${\vec{B}}_{0}$, regardless of the underlying microscopic mechanisms.

### 4.3 Limitations and future work

#### 4.3.1 Anatomical correspondence

The pooled analysis considers all single fibre population voxels throughout the WM together to estimate a single magnitude of orientation dependence; however, the simulations reveal that micro-anatomical differences (e.g., signal fractions, myelin sheath thickness, fibre density, and other potential contributors to compartmental $T_{2}$-differences) can lead to different orientation dependence. The tractometry analysis aims to address this to a certain extent by pooling voxels more locally, but with two head orientations as used in this study it remains challenging to estimate local differences in orientation dependence. More head orientations and a boost in SNR could help to further investigate this. Here, more efficient acquisition-schemes, such as ZEBRA (Hutter et al., 2018), would be beneficial to enable reasonable acquisition times. Moreover, anatomical correspondence could be further achieved by co-registering the data from the two head orientations in future work. To accomplish this, it is essential to employ a reliable registration method that can effectively handle the residual nonlinear effects. Furthermore, by pooling the data from all subjects' SFP WM voxels, we were able to compensate for low number of subjects. With more subjects, one could investigate the anisotropy w.r.t. $B_{0}$ of individual tracts and consequently provide additional anatomical information.

#### 4.3.2 Gradient nonlinearities

Another limitation potentially arises from nonlinearities of gradient fields. With the rotation of the tiltable coil the head is positioned further from the iso-centre, where gradient nonlinearities have a larger effect. This, in turn, influences the effective B-matrix, and could introduce additional variability between the non-tilted and tilted orientation. In addition to effects reported as a result of the effective B-matrix not being taken into account (Bammer et al., 2003; Guo et al., 2020; Mesri et al., 2020), if gradient nonlinearities cause the effective b-value to be higher than the imposed value, kurtosis effects may start to play a more prominent role and bias DT estimates. In the current work, we take into account the effective B-matrices, and to further minimise this potential confound we analysed a subset of the data at TE=54 ms for which a lower b-value of $1050\;\text{s}/{\text{mm}}^{2}$ was available (Fig. 1A). Figure S4 shows a comparison of the pooled- and tractometry analyses with a maximum b-value of $1500\;\text{s}/{\text{mm}}^{2}$ and $1050\;\text{s}/{\text{mm}}^{2}$. The observation of orientation dependence remained unchanged, with larger estimated absolute magnitude of anisotropy at the lower b-value for AD, RD, and FA in both analyses and also MD in the tractometry analysis.

We also considered the effect of gradient non-linearities in the estimation of the fibre direction. In this work, the first eigenvector of the DT was used, but this can be done in alternative ways and with different estimation techniques, e.g., spherical deconvolution to obtain the fODF. The reason this work opted for the current approach is that spherical deconvolution approaches typically do not take into account gradient nonlinearities (Guo et al., 2020). The DT estimation used in this manuscript does take this into account, and the estimates of the maps and fibre direction come from the same DT estimate.

#### 4.3.3 Crossing fibres

This scope of this work is limited to single fibre population voxels. Previous work has characterised $T_{2}$ per fibre population in crossing fibre voxels, for example, Reymbaut et al. (2020). In the current work, based on Tax et al. (2021), we have simulated a distribution of orientation-dispersed compartments according to a Watson distribution, where each sub-compartment (e.g., each extra-axonal zeppelin) can separately exhibit $R_{2}$-orientation dependence. This could be straightforwardly adapted to model crossing fibres, but the bundles will have to have the same relaxation properties. A recently presented abstract described estimation of such a model for multi-echo gradient-echo sequences (Sandgaard et al., 2022).

#### 4.3.4 SNR

Finally, the SNR distribution in WM can change with head reorientation. While the tiltable coil minimises differences in the coil-to-brain distance across different head orientations, SNR may still be affected due to, e.g., change in the reception efficiency of the tiltable coil as the axis of the coil is rotated away from ${\vec{B}}_{0}$, gradient non-uniformities, or $B_{0}$ shim. Previous work (Tax et al., 2021) showed that the temporal SNR (tSNR) distribution in WM globally overlapped between tilted and default orientation, and Figure S5 further investigates this per tract-segment from the tractometry pipeline. Overall, the estimated tSNR of the same location in tilted vs default orientation is distributed along the line y=x, but a global fit through tSNR measurements from all locations implies that tSNR values in the tilted position could be up to 15% lower than in the default orientation. Preliminary experiments in a phantom with the body coils for signal reception suggest that the impact of $B_{0}$ shim and gradient non-uniformities may be greater than the impact of receive coil efficiency (results not shown). While we have attempted to correct for noise bias—which can significantly impact DTI estimates (Jones & Basser, 2004)—denoising strategies could further reduce the impact of noise differences, especially at longer TE.

## 5 Conclusion

DT measures may vary up to 20% as a function of WM fibre orientation w.r.t. ${\vec{B}}_{0}$ in the scenarios investigated. Fibre orientation can be responsible for up to 7% variance in diffusion tensor measures across single fibre populations of the whole brain white matter. While potentially containing useful information on, e.g., myelination, the orientation dependence of DTI w.r.t. ${\vec{B}}_{0}$ can be an additional source of variance camouflaging the effect-of-interest in clinical research studies, particularly when the effect size is small and it is difficult to control for fibre orientation w.r.t. ${\vec{B}}_{0}$ (e.g., fetal or neonatal imaging, or when the trajectories of fibres change due to, e.g., space occupying lesions).

## Supplementary Material

Supplementary Material

## Data Availability

Data available on request due to privacy/ethical restrictions.

## Appendix A: Key findings from Tax et al. (2021)

In our previous work, we estimated the apparent $R_{2}$-values for intra- and extra-axonal compartments from the WM SFP data acquired by varying nominal b-values and TEs simultaneously. The acquisition parameters are reproduced in this work in Figure 1A. The compartmental spin-echo signals with associated apparent $R_{2}$-values were included in the compartmental model of diffusion in WM. The latter describes the signal as a convolution of the signal associated with a population of perfectly parallel fibres with a fibre orientation distribution function. The diffusion in the intra- and extra-axonal spaces was described by “stick” and “zeppelin” tensors, respectively.

We then characterised the dependence of the compartmental $R_{2}$-values on WM fibre orientation angle θ w.r.t. ${\vec{B}}_{0}$:

$$R_{2}\left(\theta \right)=R_{2,\text{iso}}+f\left(\theta \right),$$
(A1)

$$f\left(\theta \right)=R_{2,{\text{aniso}}_{1}}\cdot {\text{sin}}^{2}\theta +R_{2,{\text{aniso}}_{2}}\cdot {\text{sin}}^{4}\theta .$$
(A2)

$R_{2,\text{iso}}$ is a θ-independent isotropic component of $R_{2}(\theta )$, whereas f(θ) describes the orientation-dependent component. We allowed the corresponding anisotropic coefficients $R_{2,{\text{aniso}}_{1/2}}$ to be independent, linked, or set to zero, to achieve different variations of this generalised representation. This resulted in a set of the following five representations:

$$R_{2}\left(\theta \right)=R_{2,\text{iso}},$$
(A3)

$$R_{2}\left(\theta \right)=R_{2,\text{iso}}+R_{2,\text{aniso}}\cdot {\text{sin}}^{2},$$
(A4)

$$R_{2}\left(\theta \right)=R_{2,\text{iso}}+R_{2,\text{aniso}}\cdot {\text{sin}}^{4},$$
(A5)

$$R_{2}\left(\theta \right)=R_{2,\text{iso}}+R_{2,\text{aniso}}\cdot \left[1-\frac{1}{4}{\left(3{\text{cos}}^{2}-1\right)}^{2}\right],$$
(A6)

$$R_{2}\left(\theta \right)=R_{2,\text{iso}}+R_{2,{\text{aniso}}_{1}}\cdot {\text{sin}}^{2}\theta +R_{2,{\text{aniso}}_{2}}\cdot {\text{sin}}^{4}\theta .$$
(A7)

All of them were used to analyse the data pooled from all SFP voxels and head orientations, while only the first three were applied to analyse data, which were anatomically matched between head orientations using tractometry. We also analysed the $R_{2}$-values estimated by fitting a mono-exponential function to the data obtained at b=0 to compare them to previous studies.

**Main results from the pooled data.** Intra-axonal $R_{2}$-values were best described by the Equation A6 with the isotropic component of $12.0\;{\text{s}}^{-1}$ and the magnitude of anisotropy of $0.8\;{\text{s}}^{-1}$. The AIC of the isotropic representation (Eq. A3) was larger than that of the anisotropic representation with Δ AIC=76 and $R_{\text{iso}}=12.6\;{\text{s}}^{-1}$. Extra-axonal $R_{2}$-values were best represented by the Equation A5 with the isotropic component of $17.4\;{\text{s}}^{-1}$ and the magnitude of anisotropy of $2.4\;{\text{s}}^{-1}$. The corresponding isotropic representation was not supported with Δ AIC=838 and $R_{\text{iso}}=18.7\;{\text{s}}^{-1}$.

**Main results from the tractometry analysis.** Intra-axonal values were best supported by the isotropic representation, while extra-axonal values were best supported by ${\text{sin}}^{4}\theta$-representation with the magnitude of anisotropy of $5.1\pm 3.0\;{\text{s}}^{-1}$.
