# Supplementary material for: The impact of head orientation with respect to B0 on diffusion tensor MRI measures
Source: Imaging Neurosci (Camb). 2023 Sep 25;1:imag-1-00012. doi: 10.1162/imag_a_00012 (PMC10884544; doi:10.1162/imag_a_00012)
Supplement: Supplementary Material [file imag_a_00012-supp.pdf]

Supporting Information for the manuscript titled “The Impact of Head Orientation with Respect to B_0 on diffusion tensor MRI measures”

Elena Kleban Derek K Jones Chantal M W Tax

1 Example of $\sin^4\theta$-fit

Figure S1 shows an example of $A + B \cdot \sin^4\theta$-function fitted (dashed red line) to the analytically estimated data (solid black line). The simulated curve is for AD at $f = 0.4$ and TE $= 100$ ms (cf., Figure 2A). In this example it is evident that even analytically simulated data do not follow the exact $\sin^4\theta$-curve, yet are well enough represented by it.

2 Example of anisotropic analysis with using a smoothing spline.

Binned and averaged data as a function of θ is the most general way to obtain the anisotropic information, i.e. the magnitude of anisotropy, in particular. Pooled data were binned in $1°$-subsets, and the metric-values and θ-values were averaged per bin: black curves in Figure S2 are the averaged values and the shaded grey areas are standard deviation. For further analysis these curves require smoothing. For that we fitted smoothing splines to the averaged values (red curves in Figure S2) using the number of data points per bin as weights (blue curves in the last plot of the same figure).

3 Anisotropy analysis of pooled data using $\sin^4\theta$ function of orientation.

Orientational anisotropy was analysed for MD, AD, RD and FA at each TE by representing the values from all SFP voxels in all datasets by a function of θ, as described in the Data Analysis paragraph of section 2.1.

In Figure S3**A** DT measures are plotted as functions of fibre orientation θ w.r.t. $\vec{B}_0$ (horizontal axes), and echo time TE (columns), along with the corresponding isotropic (dashed)

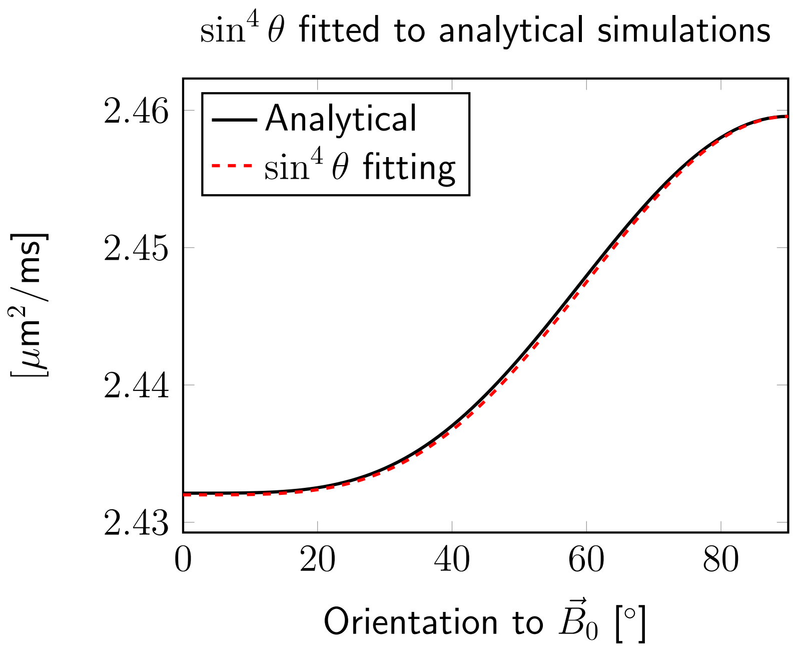

Figure S1: It is evident from the shown example of $A + B \cdot \sin^4\theta$-function fitted (dashed red line) to the analytically estimated data (solid black line) that analytically simulated data do not follow the exact $\sin^4\theta$-curve, yet are well enough represented by it.

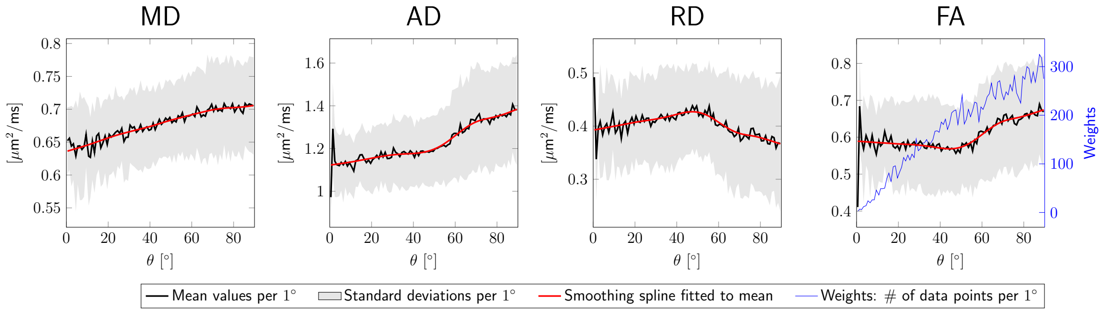

Figure S2: Smoothing spline was fitted to the data averaged within 1°-bins.

and anisotropic (solid) representation fits. The data were pooled from all subjects and both head orientations, each data point represents one SFP voxel. The barplots in Figure S3**B** show isotropic component (A, first column) or the magnitude of anisotropy (B, second column) for a given measure (rows) for isotropic and anisotropic representations (Eq. 5: $B = 0$ and $B \neq 0$, respectively). Larger ΔAIC values (third column) for the isotropic representation indicate that the measures are better represented by a function of θ with the non-zero magnitude of anisotropy.

4 Comparison between DTI measures estimated from data subsets with different maximal b-values

DTI measures were estimated for two data subsets with $b_{\text{max}} = 1500\,\text{s/mm}^2$ and $b_{\text{max}} = 1050\,\text{s/mm}^2$, respectively, at TE $= 54\,\text{ms}$. For the $b_{\text{max}} = 1500\,\text{s/mm}^2$-subset we used b-values of $[0,\ 750,\ 1500]\,\text{s/mm}^2$, and for the $b_{\text{max}} = 1050\,\text{s/mm}^2$-subset we used b-values of $[0,\ 750,\ 1050]\,\text{s/mm}^2$. Numbers of corresponding diffusion directions can be found in the table of Figure 1A. The $b_{\text{max}} = 1500\,\text{s/mm}^2$-subset corresponds to the subset used in the manuscript.

Subsequently, anisotropy analysis was performed on estimated DTI measures for pooled (Figure S4**A**) and tractometry (Figure S4**B**) data, respectively. Mean values, A, were lower for all diffusivities estimated from the $b_{\text{max}} = 1500\,\text{s/mm}^2$-subset. This is likely a direct result of kurtosis-contributors which become relevant at higher b-values. The absolute values of the magnitudes of anisotropy, $|B|$, estimated at lower maximal b-value were larger for AD, RD, and FA. In some cases they were underestimated for $b_{\text{max}} = 1500\,\text{s/mm}^2$-subset.

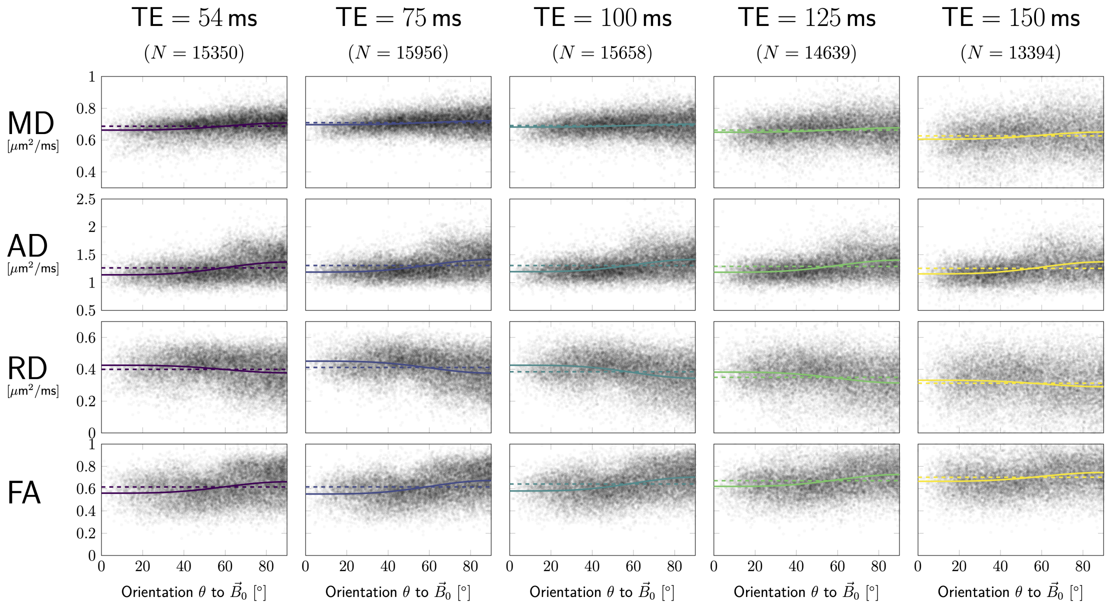

A. Each DTI measure (rows) from SFP voxels was plotted against the fibre orientation to the magnetic field θ. Each column/color corresponds to a different TE. Best-fitting isotropic (A, dashed lines) and anisotropic ($A + B \cdot \sin^4\theta$, solid lines) representations are displayed in the corresponding plots.

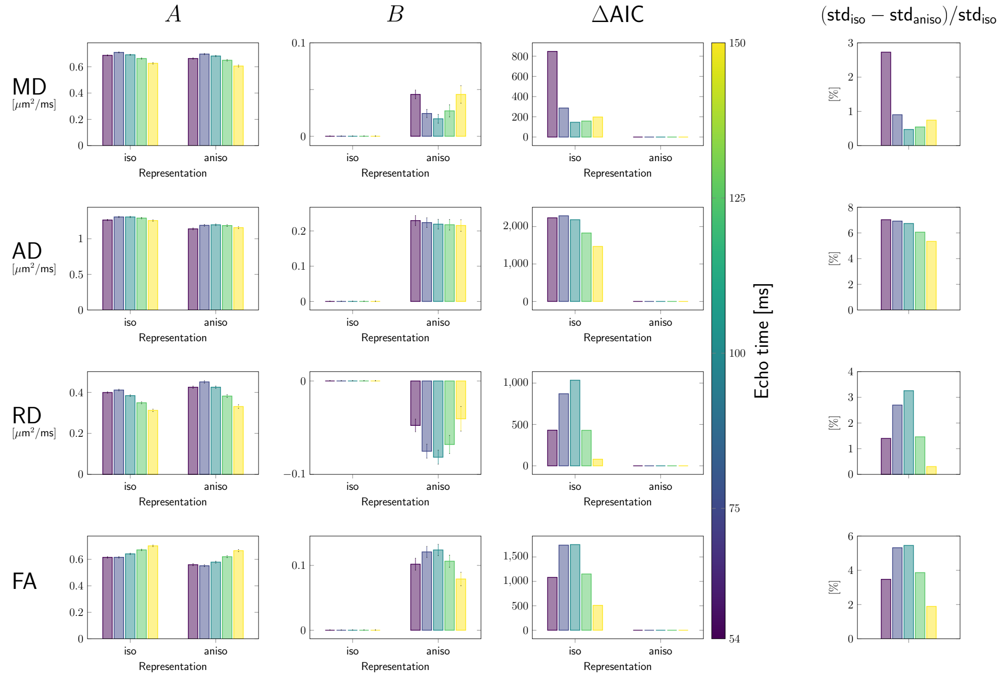

B. $A + B\sin^4\theta$, case (cf. Eq. 5). The estimated isotropic, A, and anisotropic, B, components are shown in the first and the second column, respectively. Relative values of the AIC are shown in the third column: $\Delta\text{AIC} = \text{AIC} - \text{AIC}_{\text{min}}$. Fourth column shows the amount of decrease in variation of values when orientation w.r.t. B_0 is taken into account. Colours represent the corresponding echo times, for which anisotropy of the measures wes investigated. The error bars in each barplot indicate $85\,\%$ confidence intervals.

Figure S3: **Pooled SFP data results.**

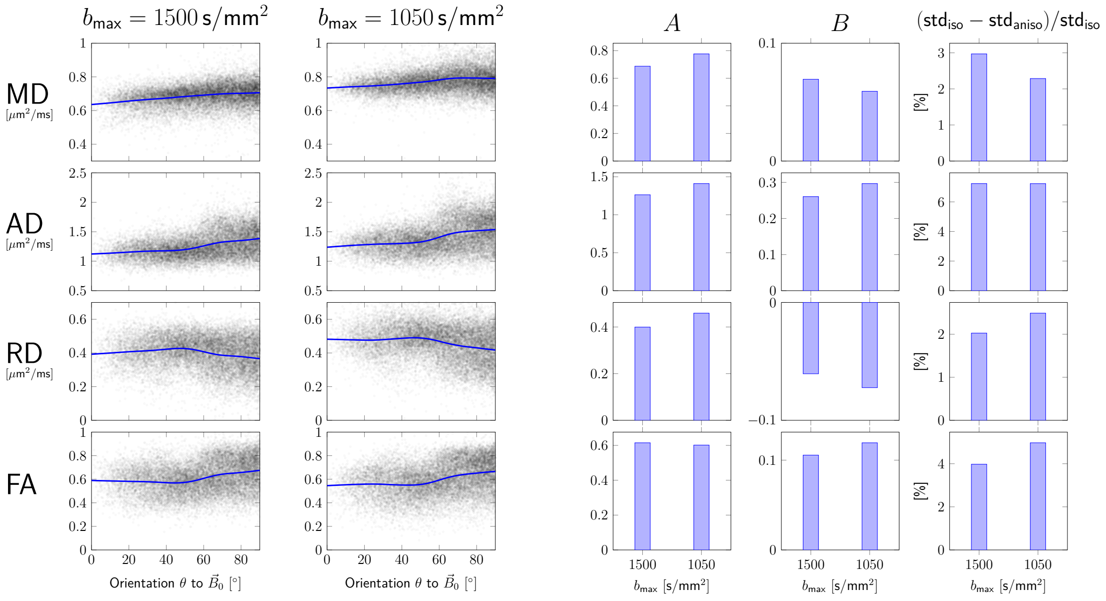

A. Pooled data.

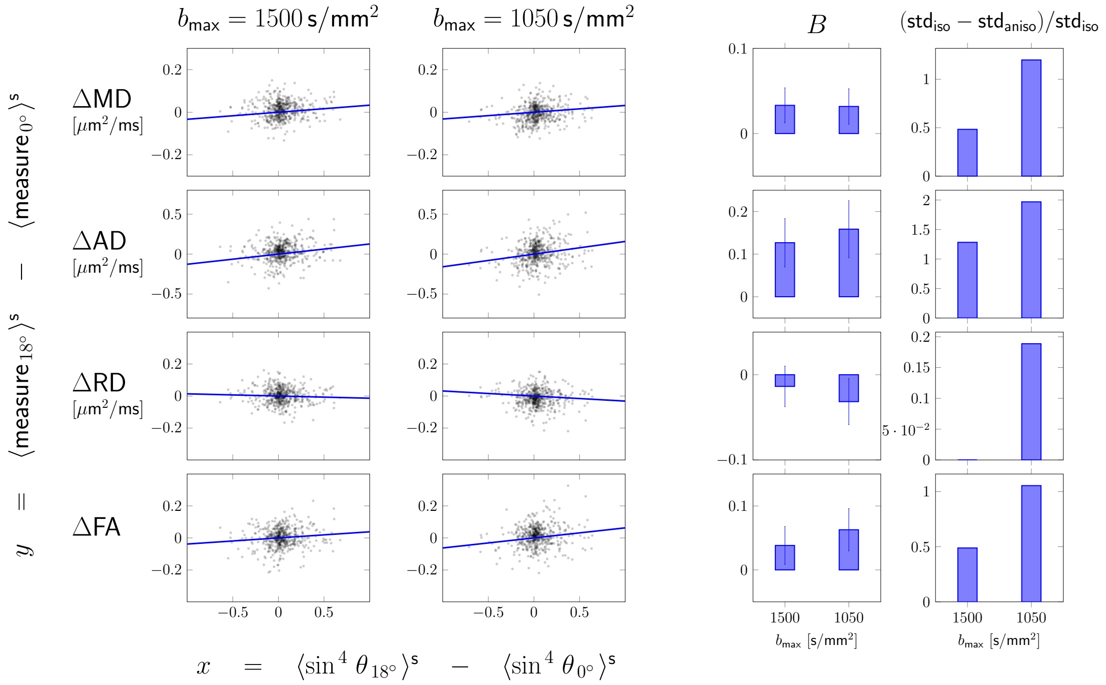

B. Tractometry data.

Figure S4: DTI measures were estimated from the subsets with two maximal b-values of $1500\,s/mm^2$ and $1050\,s/mm^2$ at TE $= 54\,ms$.

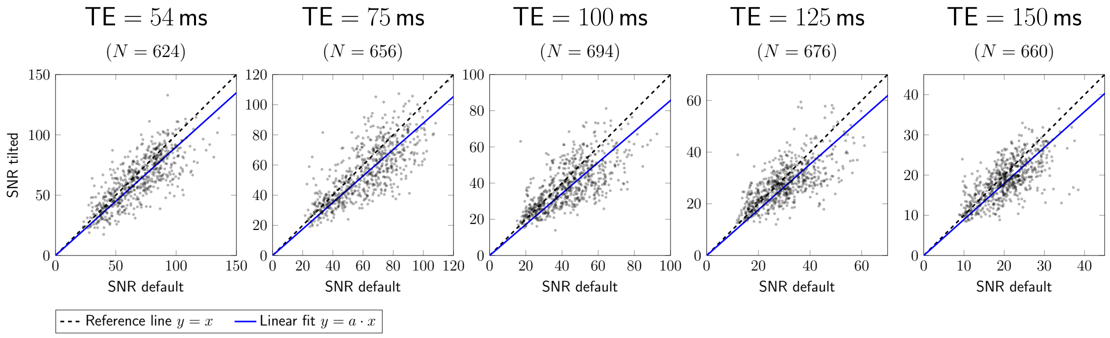

Figure S5: Temporal SNR compared for each TE between default and tilted head orientations using tract segments from the tractometry pipeline.

5 Assessing signal-to-noise ratio between tilted and default head orientations

Voxel-wise temporal signal-to-noise ratio (tSNR) estimates were obtained from the unprocessed $b = 0\,\mathrm{s/mm}^2$ images acquired at each TE by dividing the mean of the $b = 0$ images by their standard deviation. Figure S5 shows a scatter plot of the tSNR in the default position vs the tSNR in the tilted position, where each point represents the mean tSNR across all voxels in a tract-segment from the tractometry pipeline. The points are overall distributed along the line $y = x$ (the dashed black line). As an estimate of the tSNR difference, $y = a \cdot x$ was fitted to the data (solid blue line), where a implied tSNR values in the tilted position to be between 10% and 15% lower than those in the default position. A few apparent outlying points with high SNR in the default position but lower SNR in the tilted position may have affected the fit.
